# Rapid Response to Penpulimab Combined With Anlotinib and Chemotherapy in a Thoracic SMARCA4‐UT Without PD‐L1 Expression: A Case Report and Review of Literature

**DOI:** 10.1111/crj.70036

**Published:** 2024-12-08

**Authors:** Yuanhang Wang, Kelei Zhao, Jingjing Zhang, Xiaohan Yuan, Yanting Liu, Jinghang Zhang, Ping Lu, Min Zhang

**Affiliations:** ^1^ Department of Oncology The First Affiliated Hospital of Xinxiang Medical University Weihui China; ^2^ Department of Pathology The First Affiliated Hospital of Xinxiang Medical University Weihui China

**Keywords:** anlotinib, immune checkpoint inhibitors, thoracic SMARCA4‐deficient undifferentiated tumor

## Abstract

SMARCA4‐deficient undifferentiated tumor (SMARCA4‐UT) in the chest is a high‐grade malignant tumor that grows rapidly and often carries a poor prognosis. Unfortunately, there are currently no effective treatment available until now. Here, we report a case of SMARCA4‐UT in a patient who showed a swift response to a combination treatment of penpulimab, anlotinib, and chemotherapy. A 55‐year‐old man was diagnosed with thoracic SMARCA4‐UT along with metastases to multiple lymph nodes, the pleura, and bones. Immunohistochemical (IHC) testing indicated the absence of PD‐L1 expression in tumor cells. He was given sintilimab and anlotinib as first line treatment. However, a follow‐up chest CT revealed progressive disease (PD) after the first cycle treatment. Subsequently, the second line regimen was modified to etoposide and cisplatin (EP) combined with anlotinib and penpulimab. The effectiveness evaluation revealed partial remission (PR) following two cycles of the second‐line regimen treatment. Notably, the patient's progress‐free survival (PFS) exceeds 7 months and the overall survival up to 12 months. Our case implies that a combination of chemotherapy, anlotinib, and penpulimab might offer a promising therapeutic approach for PD‐L1‐negative thoracic SMARCA4‐UT.

## Graphical abstract

A 55‐year‐old male with advanced thoracic SMARCA4‐deficient undifferentiated tumor (SMARCA4‐UT) and no PD‐L1 expression achieved a durable partial response with second‐line therapy combining etoposide‐platinum chemotherapy, anlotinib, and penpulimab. Maintenance therapy and palliative radiotherapy provided temporary disease control before progression at 12 months. This case highlights the potential of multimodal therapy targeting SMARCA4 and TP53 mutations in improving outcomes for this rare, aggressive malignancy.

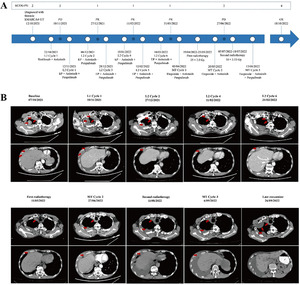

## 1 Case Report

A male patient, aged 55 years, initially sought treatment at another hospital due to complaints of pain in the right side of his chest. A CT scan conducted at the previous hospital showed an abnormal high‐density shadow in the right lung accompanied by abundant right pleural effusion. To obtain a more accurate diagnosis and commence appropriate treatment, the patient was subsequently referred to our hospital, and upon evaluation, he was diagnosed with thoracic SMARCA4‐UT on October 12, 2021, with multiple lymph node metastases and pleural and bone metastases (cT3N2M1a).

**FIGURE 1:**
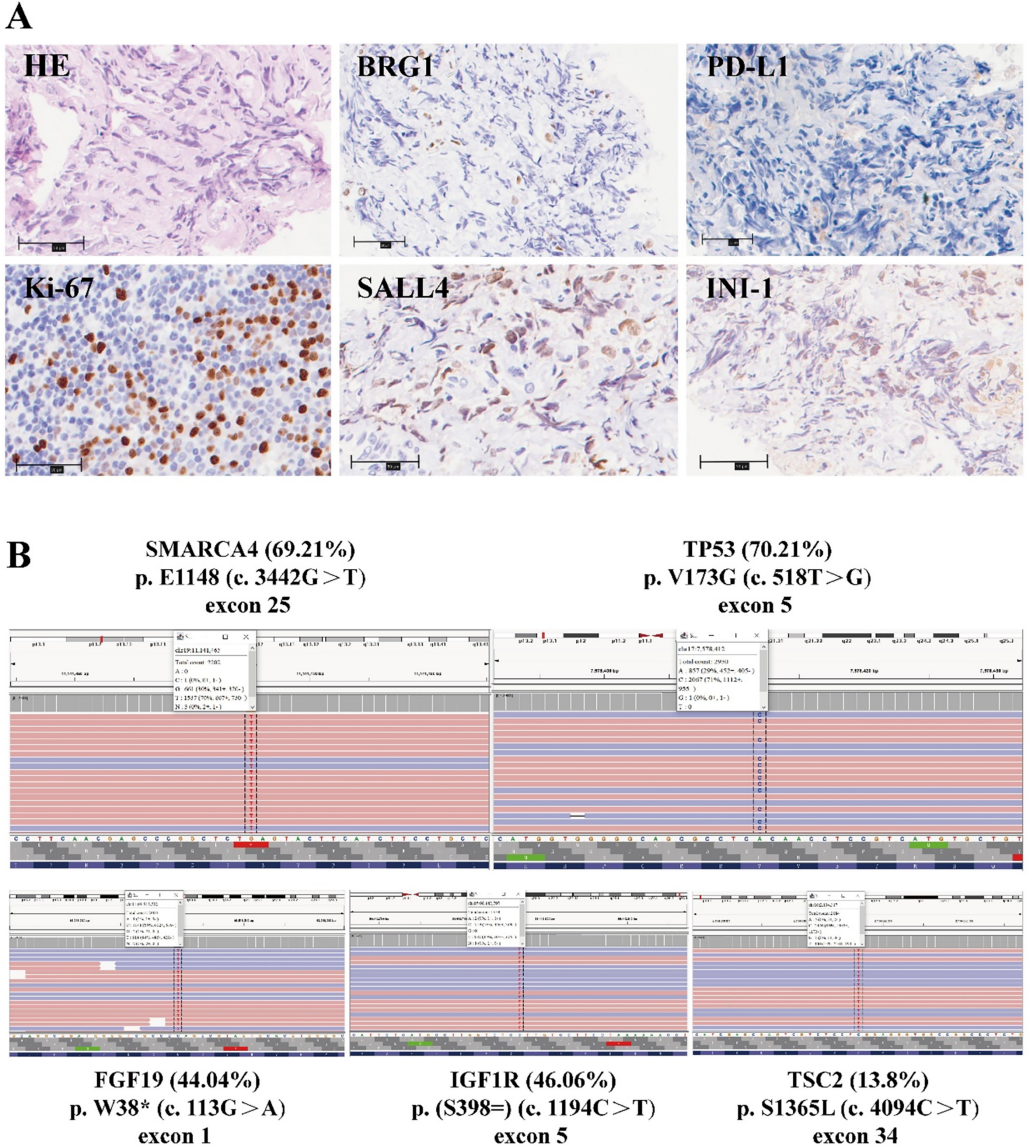
(A) Histologic and immunohistochemical features of the patient. Scale bars 50 μm. HE: hematoxylin and eosin staining revealed relatively dyscohesive singly dispersed tumor cells with eccentrically located vesicular nuclei, prominent nucleoli and eosinophilic cytoplasm; BRG1 (SMARCA4): a complete lack of BRG1 expression on tumor cells; PD‐L1: a complete lack of PD‐L1 expression on tumor cells; Ki‐67: diffusely expressed in tumor cells; SALL4: expressed in tumor cells; INI‐1 (SMARCA1): expressed in tumor cells. (B) Results of the second‐generation sequencing (NGS).

At diagnosis, the patient's complaint was persistent prickling‐like right thoracic pain. Imaging examination revealed a right pleural mass with pulmonary emphysema, lung bullae, right pleural effusion and atelectasis, right hilar lymph node and supraclavicular lymph nodes enlargement, and partial right rib bone destruction (Figures S1 and S2). A CT‐guided percutaneous pleural puncture biopsy was performed (Figure S3). The IHC staining results indicated the absence of BGR1 and PD‐L1 protein expression. Other IHC showed SALL4(+), INI‐1(+), and Ki‐67(+60%) (Figure 1A). Next‐generation sequencing (NGS) analysis revealed the presence of gene mutations, notably including SMARCA4 (E1148*) and TP53 (p.V173G) (Figure 1B). The pathological diagnosis confirmed thoracic SMARCA4‐UT tumor.

**FIGURE 2:**
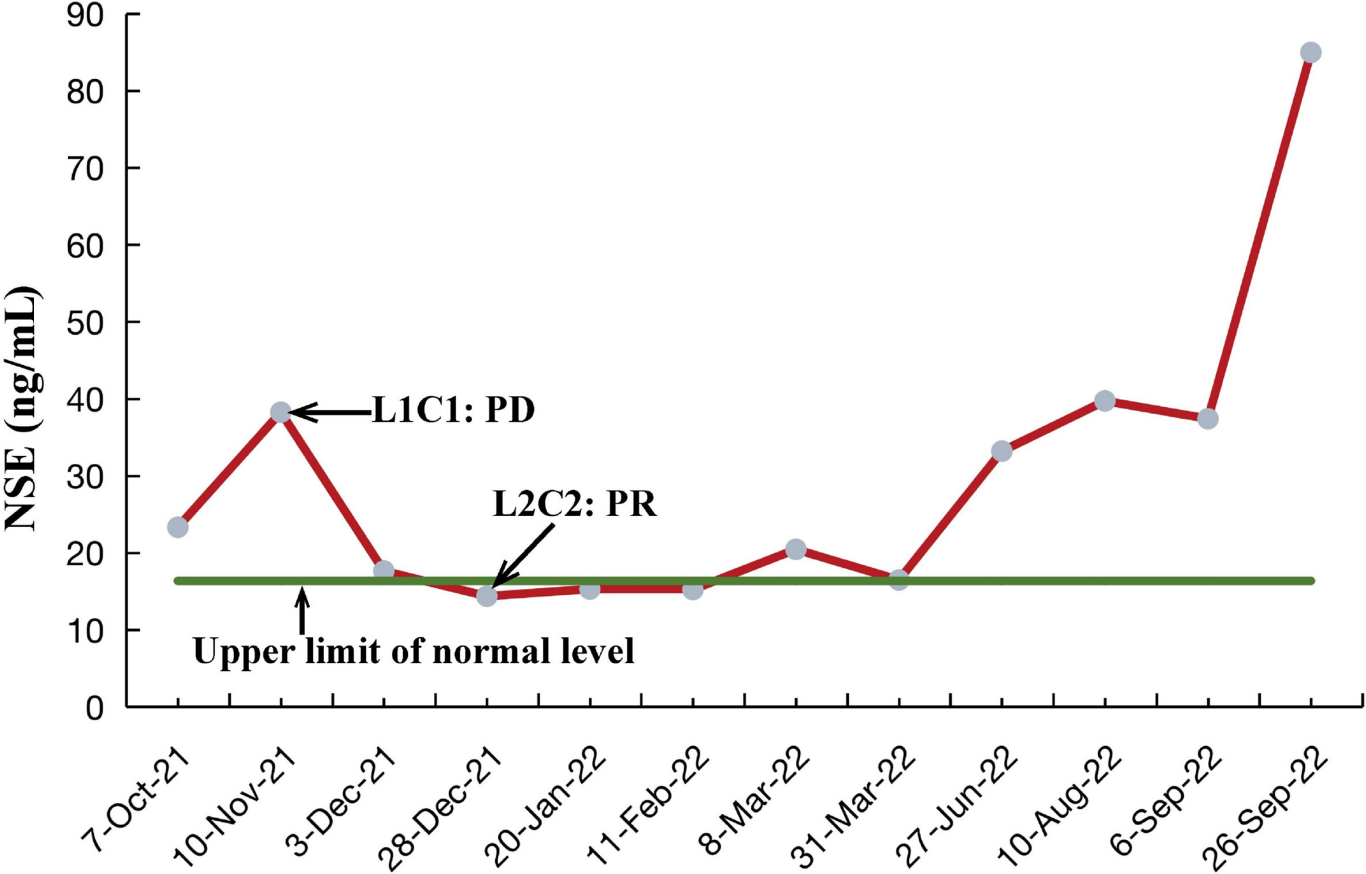
Changes in neuron‐specific enolase (NSE, normal range: 0–16.36 ng/mL) during the treatment. Green: upper limit of normal level. Red: NSE reexamination results after treatment.

**FIGURE 3:**
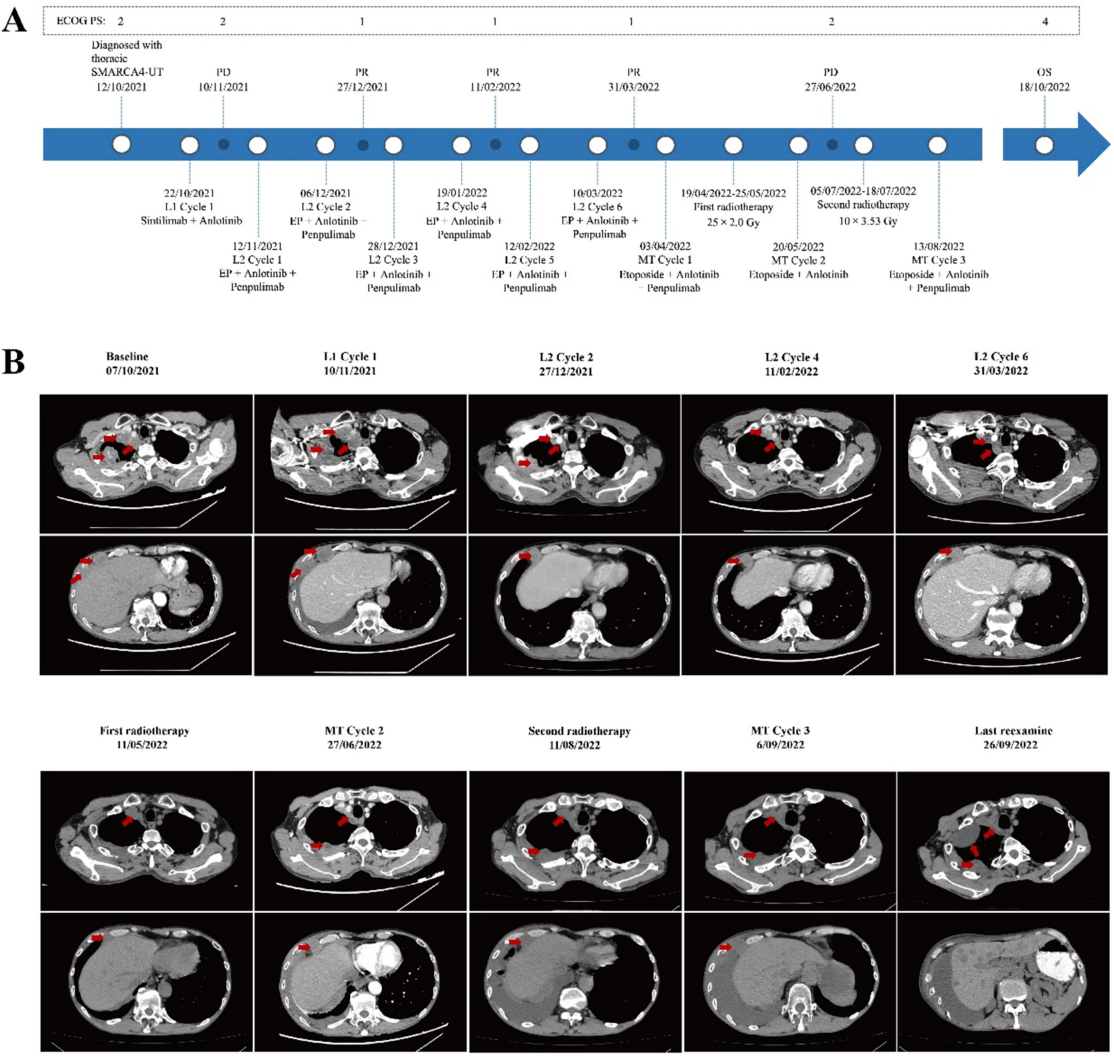
(A) Timeline of the case and Eastern Cooperative Oncology Group Performance Status (ECOG PS) score. (B) CT scan follow‐up. Changes in transverse CT reexamination images of the chest during the treatment. Baseline: patient admission diagnostic examination; L1 cycle 1: after the first‐line therapy. L2 cycle 4: after the fourth cycle of second‐line therapy; L2 cycle 6: after the sixth cycle of second‐line therapy; first radiotherapy: after the first radiotherapy; MT cycle 2: after the second cycle of maintenance treatment; second radiotherapy: after the second radiotherapy; MT cycle 3: after the third cycle of maintenance treatment; last reexamine: last CT reexamination before death.

The patient received a combination regimen of sintilimab and anlotinib as the first‐line treatment. Before the second treatment cycle, the patient experienced worsening chest pain. Subsequently, chest CT scans showed an increase in lesions within the right and interlobar pleural area, larger hilar lymph nodes, and increased pleural effusion (Figure 3B), the efficacy evaluation was PD. Then, based on the results of the NGS (Figure 1B), the second‐line therapy regimen was modified to include a combination of EP with anlotinib and penpulimab. The treatment adjustment was initiated on November 12, 2021, and continued until March 15, 2022. After the second‐line two‐cycle treatment, neuron‐specific enolase (NSE) showed a decline from a peak of 38.25 ng/mL to a nadir of 14.37 ng/mL (Figure 2). The chest pain was resolved. And ECOG PS score dropped to 1 (Figure 3A). Strikingly, chest CT scan demonstrated the markedly shrinkage of the chest mass and mediastinal lymph nodes, with a concomitant dramatic volumetric reduction of pleural effusion, and the efficacy evaluation was PR (Figure 3B). However, the destruction of the right eighth rib was slightly more obvious than before. Following six cycles of the second‐line treatment, a CT scan revealed a sustained and durable PR response without any reported adverse events (Figure 3B). But metastases showed mild thickening of the right pleura and mild enlargement of the mediastinal lymph nodes. As of March 15, 2022, the patient has been on an EP regimen combined with anlotinib and penpulimab for 4 months.

In April 2022, the patient proceeded to receive three cycles of maintenance treatment (MT), which involve the combination of etoposide capsules combined with anlotinib and penpulimab (the second cycle without penpulimab). After two cycles of MT, a follow‐up chest CT examination revealed a thickening of the pleura. During maintenance treatment, the patient received one TOMO chest radiotherapy (25 × 2.0 Gy) and one chest metastatic tumor radiotherapy (10 × 3.5 Gy) to alleviate pain (Figure S4). After the first radiotherapy sessions, the pleural tumor was significantly reduced. But there are new nodules in the upper lobes of both lungs. After the second radiotherapy, the solid component of the tumor was smaller than before, but the nodules of the upper lobe of the right lung were slightly larger than before.On September 26, 2022, a chest CT reexamination indicated tumor progression along with multiple liver metastases (Figure 3B, last reexamine). At this stage, further anti‐tumor treatments were rejected by patients and their families, and the patient was given palliative and symptomatic treatment. The patient was followed up regularly until October 2022 while receiving antitumor therapy. The follow‐up process is illustrated in Figure 3A. The patient's condition deteriorated, and he died on October 18, 2022. The overall survival (OS) was 12 months.

## 2 Discussion

Thoracic SMARCA4‐deficient undifferentiated tumor was initially described in 2015 [1]. Subsequently, in April 2019, a comprehensive review of the clinicopathologic features of 30 cases and proposed the pathological diagnosis criteria of SMARCA4‐UT was presented for the first time [2]. Building upon this work, the World Health Organization (WHO) presented a complete diagnostic criteria in 2021 [3].

However, there is still no standard treatment option to date, and the prognosis is extremely poor, with a median OS of 7 months in advanced patients. The management mostly involves systemic and aggressive local therapies, even in cases where metastases occur [2, 4, 5, 6, 7]. Importantly, the majority of cases are diagnosed in advanced disease stages while conventional radiotherapy and chemotherapy have shown limited success in improving disease prognoses [2, 5, 6, 7, 8, 9, 10, 11, 12, 13, 14]. Immune checkpoint inhibitors (ICIs) or immune combination therapy has shown favorable responses; however, not all patients benefit equally (Table 1). The presence of PD‐L1 in tumor cells is a significant biomarker that could indicate the potential response to anti‐PD‐1/PD‐L1 agents [15]. Patients with SMARCA4‐UT who exhibit high PD‐L1 expression (≥ 50%) are appropriate candidates for ICI therapy [16, 17, 18, 19]. Interestingly, some but not all cases with low or negative PD‐L1 expression have also been shown to respond well to ICIs [5, 15, 19, 20, 21, 22]. This aligns with the case we describe here as the patient lacked PD‐L1 expression, yet his PFS exceeded 7 months and his OS exceeded the median OS observed in SMARCA4‐UT patients.

**TABLE 1:** Cases of SMARCA4‐UT with immune checkpoint inhibitor.

Cases	Sex	Age(y)	PD‐L1 expression	Treatment	Outcome	Vital status (times)
Case (1) Henon et al. (2019)	F	58	Negative (0%)	Pembrolizumab	PR (72%)	NA
Case (2) Kunimasa et al. (2019)	M	45	Negative (0%)	ICI	NA	DOD (11 months)
	M	45	Negative (0%)	CBDCA + nab‐PTX + ICI	NA	DOD (11 months)
Case (1) Iijima et al. (2020)	M	76	Negative (< 1%)	Nivolumab	A dramatic regression of tumor size to an almost undetectable level	AWD (22 months)
Case (1) Takada et al. (2020)	F	70	High (60%)	Pembrolizumab	PR	NA
Case (1) Anžič et al. (2021)	M	41	High (100%)	Ipilimumab + pembrolizumab	A mixed response	DOD (26 months)
Case (1) Lriguchi et al. (2021)	M	50s	NA	CBDCA and nab‐PTX + Atezolizumab	SD, but right adrenal metastasis progressed	NA
Cases (3) Kawachi et al. (2021)	F	73	Intermediate (40%)	Atezolizumab + bevacizumab + PTX + CBDCA	PR (30%)	NA
	M	59	Negative (0%)		PR (59%)	NA
	F	64	High (80%)		PR (30%)	NA
Case (1) Kunimasa et al. (2021)	M	51	Negative (0%)	Atezolizumab + bevacizumab + PTX + CBDCA	The tumor shrank by 6 cm	NA
Case (1) Nambirajan et al. (2021)	M	41	High (100%)	Palliative chemotherapy + pembrolizumab + ipilimumab	PR	DOD (22 months)
Case (1) Tanaka et al. (2021)	M	58	Intermediate (10%)	Pembrolizumab + CBDCA + PEM	Significant tumor shrinkage comparable to partial response	AWD (11 months)
Cases (4) Gantzer et al. (2022)	M	40	Negative (0%)	ICI	No response	DOD (1.2 months)
	F	66	Negative (0%)	Nivolumab	No response	DOD (2.2 months)
	M	39	Negative (< 1%)	Ipilimumab + nivolumab	Rapid and partial long‐term response	Alive (NA)
	M	69	Negative (0%)	ICI	No response	DOD (6.5 months)

Abbreviations: AWD, alive with disease; CBDCA, carboplatin; DOD, died of disease; F, female; ICI, immune checkpoint inhibitor; M, male; NA, data not available; PEM, pemetrexed; PR, partial response; PTX, paclitaxel; RT, radiation therapy; SD, stable disease; y, years.

After reviewing the relevant literature, it was considered that the effectiveness of chemotherapy was poor. In addition, anlotinib targets include FGFR/CDK4, among others, and is effective against lung squamous cell carcinoma, lung adenocarcinoma, and small cell lung cancer. Therefore, we used anlotinib in combination with sintilimab as first‐line treatment. But first‐line treatment does not work well. Subsequently, we adopted second‐line treatment regimen for the following reasons: (1) to predict potential therapeutic targets based on genes; (2) neuroendocrine markers were significantly elevated (Figure 2); (3) in this case, the characteristics of SMARCA4‐UT are more inclined to small cell lung cancer. To be specific, the patient has gene mutations, including *SMARCA4* (E1148*), *TP53* (p.V173G), and *DYNC2H1*. As we all know, *TP53* gene mutation patients can benefit from chemotherapy. Patients with concurrent *TP53* and *DYNC2H1* mutations have shown improved efficacy toward etoposide and platinum drug treatments [23, 24]. Furthermore, the presence of a TP53 mutation could potentially predict a positive response to immunotherapy as it has been shown to enhance the expression of immune checkpoints, activate T‐effector cells, and stimulate interferon‐γ‐signature [25]. Studies have demonstrated significant clinical benefits of immune checkpoint inhibitors (ICIs) in patients with *TP53* mutations [25, 26]. Besides, anlotinib is a small molecule multi‐target tyrosine kinase inhibitor (TKI), which has shown potential in overcoming chemotherapy resistance by inhibiting FGFR1‐4 activation [27, 28]. *SMARCA4* mutations are associated with multiple signaling pathway abnormalities (e.g., MAPK, Wnt, and Notch pathways), and inhibiting key targets of these pathways (e.g., FGFR and VEGFR) with targeted drugs (e.g., anlotinib) may help reverse the malignant phenotype of tumors [29, 30]. A combination of immunotherapy, chemotherapy, and targeted therapy was used in this case. Chemotherapy can induce immunogenic cell death (ICD), release tumor antigens, stimulate the body's anti‐tumor immune response, and enhance the effect of immunotherapy. At the same time, by inhibiting angiogenesis, anlotinib may also increase the infiltration of T cells in the tumor microenvironment, further enhancing the response to immunotherapy [25]. In addition, *TP53* mutations themselves increase the tumor mutation load and produce more tumor antigens, which has a synergistic effect with the mechanism of immunotherapy. The mechanism hypothesis can be further verified by multi‐omics analysis, animal models, and organoid models in the future.

Therefore, the presence of *SMARCA4* and *TP53* mutations may influence tumor biological behavior and treatment sensitivity through multiple mechanisms, including altering the immune response, enhancing sensitivity to chemotherapy drugs, and overcoming drug resistance by inhibiting key signaling pathways through targeted therapy. These mechanisms interact to enable the combination therapy to produce a more significant anti‐tumor effect.

In conclusion, we report a rare case of rapid response to a treatment regimen consisting of penpulimab combined with anlotinib and chemotherapy in a patient with thoracic SMARCA4‐UT lacking PD‐L1 expression. Although the effectiveness warrants further evaluation and the underlying treatment mechanism needs expansive investigation to enrich our understanding, the treatment regimen described here holds potential as a valuable therapeutic option for SMARCA4‐UT in the future.

## Author Contributions

The first draft of the manuscript was written by Yuanhang Wang and Kelei Zhao. Material preparation and data collection were performed by Jingjing Zhang, Xiaohan Yuan, Yanting Liu, and Jinghang Zhang; writing – review and editing was performed by Ping Lu and Min Zhang.

## Conflicts of Interest

The authors declare no conflicts of interest.

## Supporting information


**Figure S1** Chest CT plain scan and enhanced scan. (a) and (b) Multiple nodular and lumpy high‐density images were observed in the right pleura and interlobar pleura, and the enhanced scan showed uneven enhancement; (c) Enlarged lymph node shadow can be seen in the right pulmonary hilum with uneven enhancement; (d) Partial destruction of ribbone adjacent to the tumor site.

**Figure S2** Supraclavicular lymph node color ultrasound. Several heterogeneous echoes can be detected in the right suprasosseous fossa, with poorly defined boundaries, irregular shape, and colored blood flow signals.

**Figure S3** Biopsy by puncture. (a) right pleural puncture mass; (b) HE staining.

**Figure S4** Radiotherapy target. (a) Before the first radiotherapy; (b) After the first radiotherapy treatment; (c) Before the second radiotherapy; (d) After the second radiotherapy.

## Data Availability

The data that support the findings of this study are available from the corresponding author upon reasonable request.
