# Supplementary figures and images for: Rapid Response to Penpulimab Combined With Anlotinib and Chemotherapy in a Thoracic SMARCA4‐UT Without PD‐L1 Expression: A Case Report and Review of Literature
Source: Clin Respir J. 2024 Dec 8;18(12):e70036. doi: 10.1111/crj.70036 (PMC11625506; doi:10.1111/crj.70036)

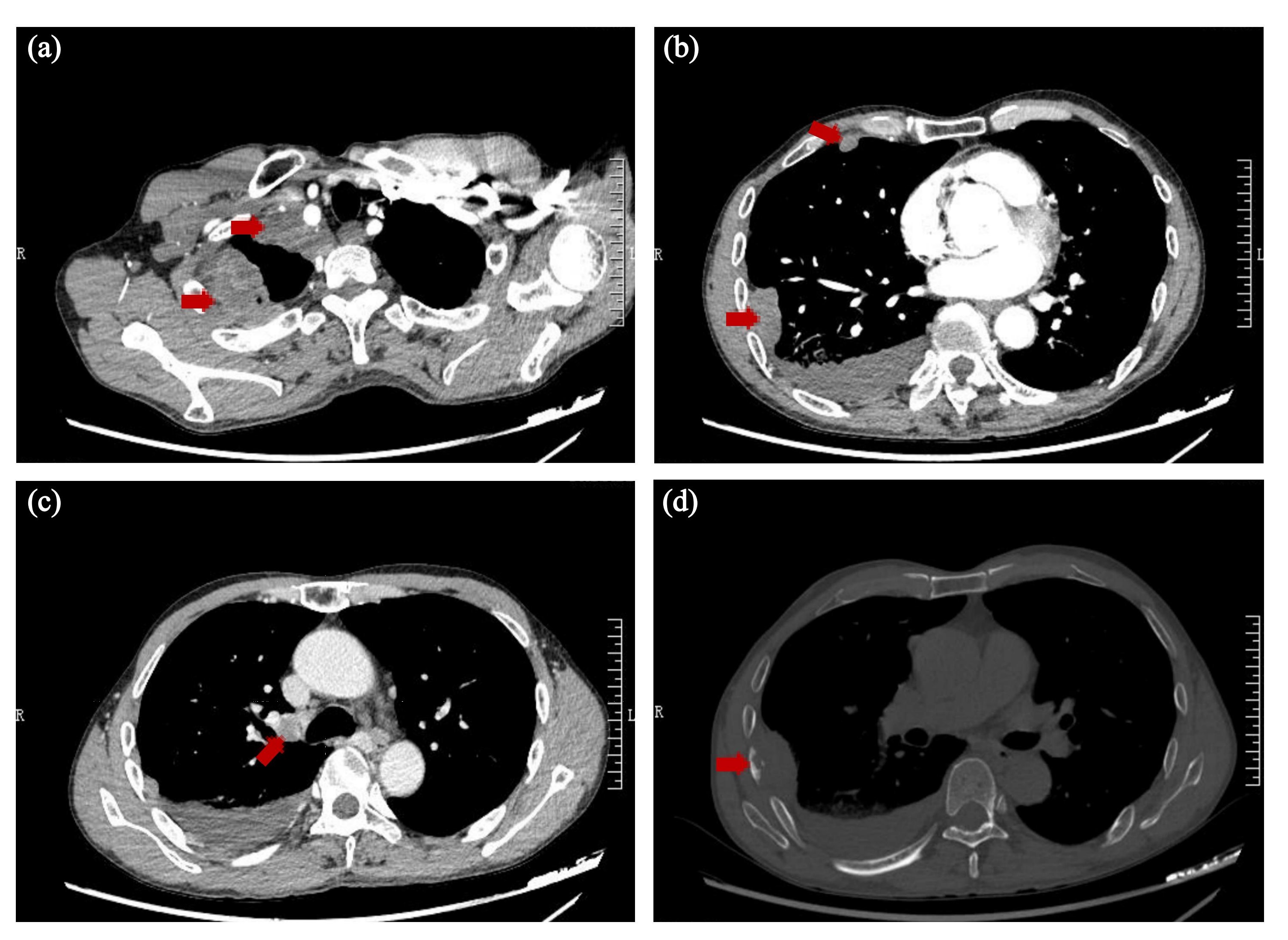

Supplement: Supplementary file 1 — Figure S1 Chest CT plain scan and enhanced scan. (a) and (b) Multiple nodular and lumpy high‐density images were observed in the right pleura and interlobar pleura, and the enhanced scan showed uneven enhancement; (c) Enlarged lymph node shadow can be seen in the right pulmonary hilum with uneven enhancement; (d) Partial destruction of ribbone adjacent to the tumor site. [file CRJ-18-e70036-s002.jpg]

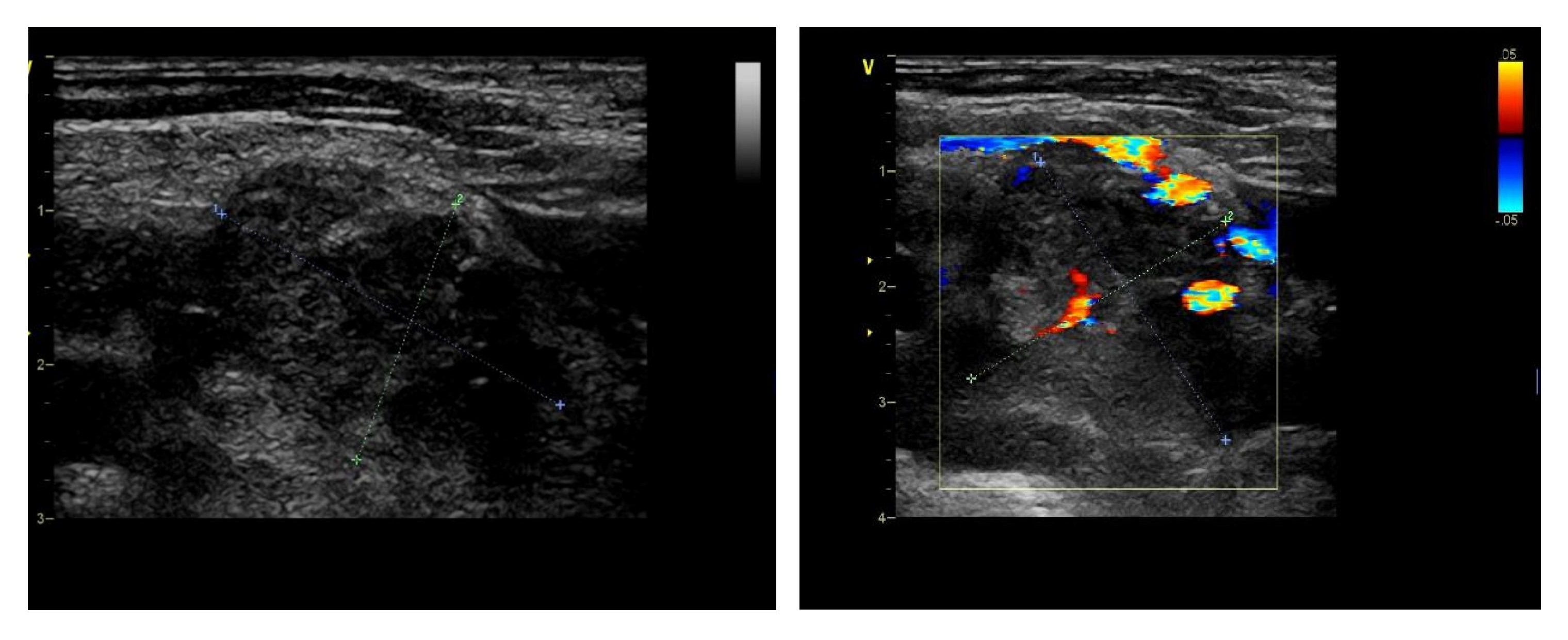

Supplement: Supplementary file 2 — Figure S2 Supraclavicular lymph node color ultrasound. Several heterogeneous echoes can be detected in the right suprasosseous fossa, with poorly defined boundaries, irregular shape, and colored blood flow signals. [file CRJ-18-e70036-s003.jpg]

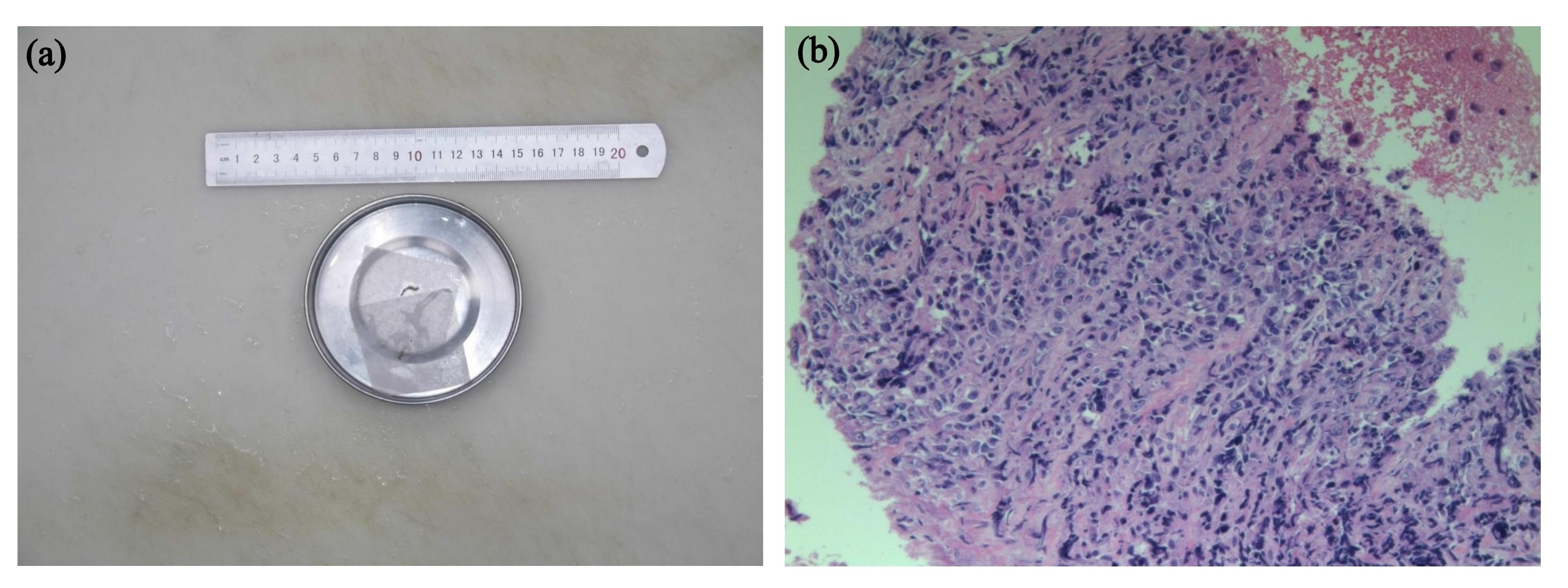

Supplement: Supplementary file 3 — Figure S3 Biopsy by puncture. (a) right pleural puncture mass; (b) HE staining. [file CRJ-18-e70036-s001.jpg]

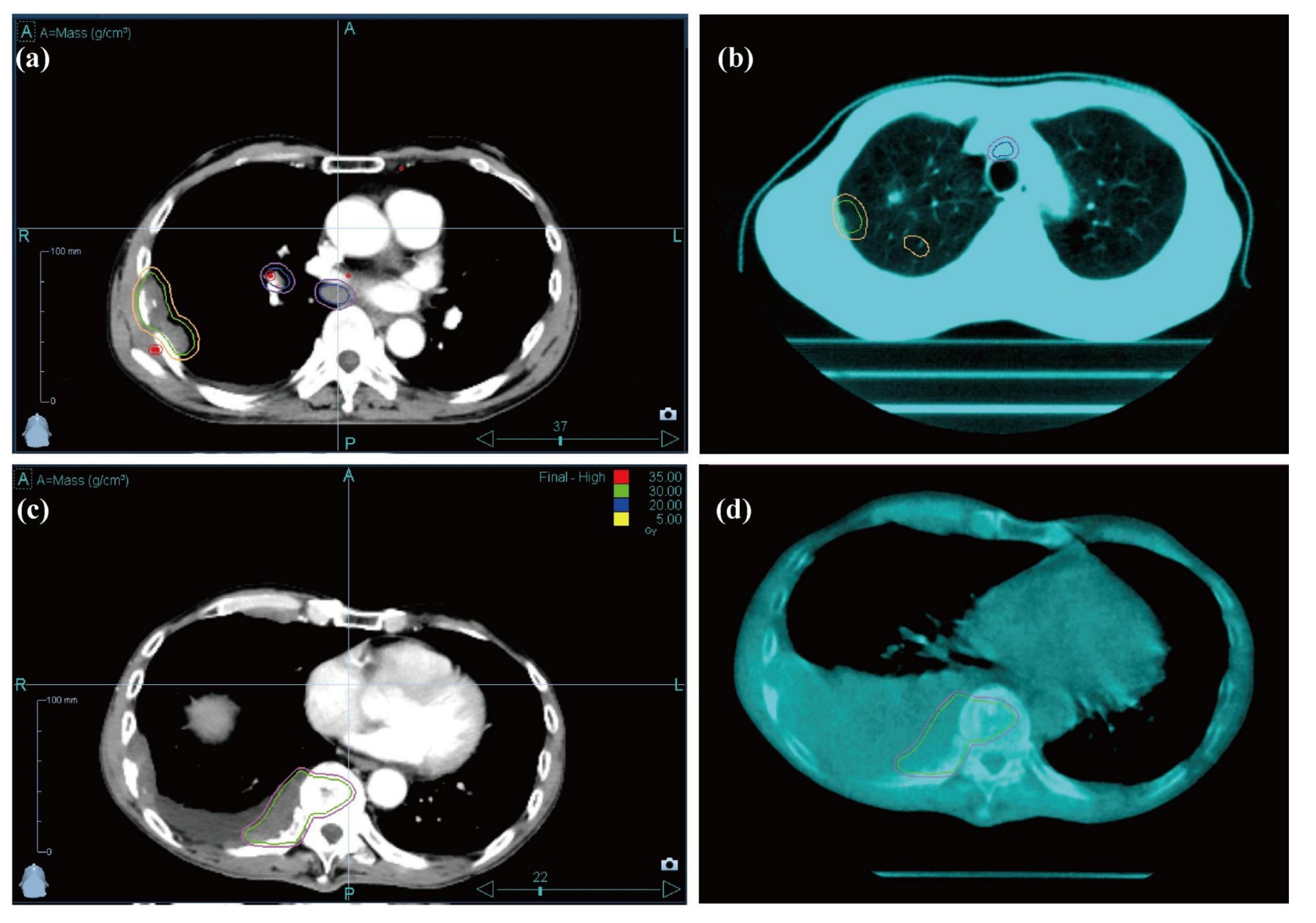

Supplement: Supplementary file 4 — Figure S4 Radiotherapy target. (a) Before the first radiotherapy; (b) After the first radiotherapy treatment; (c) Before the second radiotherapy; (d) After the second radiotherapy. [file CRJ-18-e70036-s004.jpg]
